# COHORT DIFFERENCES IN THE IMPORTANCE OF SEXUALITY AND EVALUATION OF ONE’S SEX LIFE IN LATE MIDLIFE

**DOI:** 10.1093/geroni/igz038.2919

**Published:** 2019-11-08

**Authors:** Karolina Kolodziejczak, Johanna Drewelies, Dorly J Deeg, Martijn Huisman, Denis Gerstorf

**Affiliations:** 1 Humboldt University Berlin, Berlin, Berlin, Germany; 2 Amsterdam UMC, Vrije Universiteit Amsterdam, Amsterdam, Noord-Holland, Netherlands; 3 Vrije Universiteit Amsterdam, Amsterdam, Noord-Holland, Netherlands

## Abstract

Age-related declines in multiple aspects of sex life are well documented, but we know little about historical change in key sexuality facets. We examine cohort differences in the perceived importance of sexuality and the evaluation of one’s sex life among middle-aged adults. We compare data from 55 to 64-year-olds in the Longitudinal Aging Study Amsterdam (LASA) obtained in 1992–1993 (n = 718) vs. 2012–2013 (n = 860). Results revealed that later-born adults perceive sexuality as more important than their earlier-born peers. Effect sizes were small at the sample level (d < .15), but substantial for particular subpopulations (women without partner: d = .56). In zero-order models, later-born adults evaluated their sex life as slightly less pleasant, but differences did not hold when covarying relevant individual and cohort difference factors. We conclude that historical changes in late-midlife sexuality are multifaceted and discuss theoretical and practical implications of our findings.

